# Isolation and characterization of a novel lytic bacteriophage vB_EcoS_P78 against multidrug-resistant *Escherichia coli*

**DOI:** 10.1016/j.virusres.2025.199652

**Published:** 2025-10-29

**Authors:** Rong Wen, Jiahao Tong, Shoude Liu, Chengbo Zheng, Jinshui Zheng, Donghai Peng, Ming Sun

**Affiliations:** a National Key Laboratory of Agricultural Microbiology, Huazhong Agricultural University, Wuhan 430070, Hubei, China; b National Engineering Research Center of Microbial Pesticides, Huazhong Agricultural University, Wuhan 430070, China; c College of Life Science and Technology, Huazhong Agricultural University, Wuhan 430070, China; d Hubei Hongshan Laboratory, Wuhan 430070, China; e Hubei Key Laboratory of Agricultural Bioinformatics, College of Informatics, Huazhong Agricultural University, Wuhan 430070, China

**Keywords:** *Escherichia coli*, Bacteriophage, Genomic characteristics, *Siphovirus*, Multidrug-resistant (MDR), Diarrhea

## Abstract

*Escherichia coli* phage vB_EcoS_P78, a novel bacteriophage isolated from swine feces, exhibits typical siphovirus morphology with an isometric head and a non-contractile tail, forming clear plaques approximately 4 mm in diameter. It demonstrated a broad host range, lysing 75 % of tested ESBL-producing and MDR *E. coli* clinical isolates across multiple serogroups. The phage displayed strong stability, retaining activity at temperatures up to 50 °C and within a pH range of 3–10. Results from one-step growth assays showed a 20-minute latent period and that each infected cell produced a burst size of 220 PFU. Whole-genome sequencing revealed a 44,574 bp dsDNA genome containing 58 ORFs, with no antibiotic resistance, and virulence genes detected. Phylogenetic analysis based on intergenomic similarity and core protein phylogeny consistently supported its classification within the genus *Dhillonvirus*, showing 92.8 % similarity to *Escherichia* phage JL1. These results indicate that vB_EcoS_P78 represents a novel species of *Dhillonvirus* with potential therapeutic applications against multidrug-resistant *E. coli*.

## Highlights

• A novel lytic bacteriophage vB_EcoS_P78 targeting multidrug-resistant *Escherichia coli* is isolated and identified as a new species.
• Systematic characterization reveals the basic biological properties of phage vB_EcoS_P78 with distinct lytic activity against diarrhea-associated pathogenic strains in vitro.
• As a novel foundational microbial resource, this phage enables the development of phage-based control for piglet colibacillosis diarrhea and provides a promising alternative against worsening multidrug resistance in swine pathogenic bacteria.

## 1 Introduction

*E. coli* is a Gram-negative, facultative anaerobe commonly found in the lower intestines of warm-blooded animals, such as humans, as a commensal organism. While most strains are non-pathogenic and maintain intestinal homeostasis, some possess virulence traits that enable them to induce a range of diseases (Kaper et al., 2004). Pathogenic variants are classified into several major pathotypes, including Enterotoxigenic *E. coli* (ETEC), Enteropathogenic *E. coli* (EPEC), Enterohemorrhagic *E. coli* (EHEC), Enteroaggregative *E. coli* (EAEC) and Uropathogenic *E. coli* (UPEC) based on distinctive molecular mechanisms, clinical manifestations, and serological profiles (Denamur et al., 2021). Although these pathotypes share several common mechanisms for intestinal colonization and pathogenesis, they differ substantially in their disease progression, onset and complications (Croxen et al., 2013). These pathotypes employ diverse strategies to colonize host tissues and evade immune responses, leading to a spectrum of illnesses ranging from self-limiting gastroenteritis to life-threatening hemolytic uremic syndrome and septicemia (Croxen et al., 2013). The treatment of *E. coli* infections poses considerable clinical challenges, largely due to the rising prevalence of multidrug-resistant strains, especially extended-spectrum β-lactamase (ESBL)-producing isolates, which severely limit therapeutic options. Conventional antibiotic regimens are increasingly undermined by resistance genes and biofilm-mediated protection (Singh et al., 2017), emphasizing the pressing demand for innovative therapeutic approaches.

With pathogenic *E. coli*'s increasing resistance to antimicrobials becoming a greater challenge, bacteriophage (phage) therapy has resurfaced as a viable therapeutic option with strong potential. As naturally occurring bacterial viruses, phages possess the ability to specifically target and lyse antibiotic-resistant strains, including ESBL-producing and biofilm-forming *E. coli* (Lin et al., 2017). The key advantages of phage therapy include its high specificity, which minimizes damage to the commensal microbiota (Loc-Carrillo and Abedon, 2011), and its capacity for self-replication at infection sites, enabling sustained antibacterial activity with low initial doses (Sulakvelidze and Morris, 2001). Furthermore, synergistic effects with conventional antibiotics can enhance treatment efficacy and decrease the risk of resistance emerging (Torres-Barcelo and Hochberg, 2016). Nevertheless, the clinical translation of phage therapy faces several challenges. Its narrow host range often requires customized phage cocktails to target diverse bacterial strains (Nobrega et al., 2018), while rapid emergence of bacterial resistance and potential immune neutralization pose additional hurdles (Roach et al., 2017). Current strategies to overcome these limitations include isolating novel phages from environmental sources to broaden therapeutic options (Amr et al., 2025), engineering phages to expand host range and enhance biofilm penetration (Lemire et al., 2018), optimizing phage-antibiotic combinations (Tagliaferri et al., 2019), and developing encapsulation systems to evade immune detection (Malik et al., 2017).

In this study, a novel bacteriophage was recovered from porcine fecal samples and comprehensively characterized. We evaluated its key biological properties and determined its complete genome sequence. Comparative genomic analysis was conducted to elucidate its phylogenetic relationships and functional gene profile relative to other known phages. These findings provide insights into its potential application as a biocontrol agent against pathogenic *E. coli*.

## 2 Materials and methods

### 2.1 Bacterial strain and culture conditions

All bacterial strains utilized in the study, as well as their respective characteristics, are summarized in Table 1, and the strain was provided by the Key Laboratory of Preventive Veterinary Medicine, Hubei Province, Huazhong Agricultural University. *E. coli* strain A78 was used as the phage screening and amplification strain and the remainder were used for phage host range determination. The LB broth medium was inoculated at 37 °C under aerobic conditions with all the strains.

**Table 1:** The host range of *Escherichia coli* phage vB_EcoS_P78.

Strain	Resistance	Origin	Antigen	MLST	Lytic activity
*E. coli* A78	ESBL	lung	O55	ST58	+
*E. coli* A132	ESBL	lung	O11	ST354	+
*E. coli* A236	ESBL	lung	O1	ST354	+
*E. coli* A423	ESBL	lung	O89	ST617	-
*E. coli* A385	ESBL	lung	O131	ST410	-
*E. coli* A343	MDR	lung	O8	ST10	+
*E. coli* A505	MDR	lung	O3	ST542	+
*E. coli* A492	MDR	lung	O3	ST542	+

**Spot-like lysis of bacteria induced by phages:**.

+ indicates the production of a lytic zone, - indicates the absence of a lytic zone. Assays were performed with three independent biological replicates, with each measured in triplicate. The resistance profile was determined according to whether a clear lytic zone was formed and the size of the zone.

### 2.2 Phage isolation and purification

In this study, bacteriophages were screened using fecal samples. These samples were collected from 28-day-old weaned piglets (Sus scrofa domesticus), with genders randomly selected. The first step was to add 5–10 g of fresh fecal samples to a sterile physiological saline solution (0.9 % NaCl) for subsequent processing. The sample was homogenized with this solution and kept static at 4 °C for one night. Then, the mixture was filtered twice through two pieces of cheesecloth and the combined filtrate was centrifuged at 10,000 × *g* for 10 min at room temperature. The supernatant was then filtered with a 0.22-μm pore size filter (Millex syringe filter, Merck-Millipore), and stored in a sterile 50-mL tube. The presence of bacteriophages was detected via a revised spot assay as specified previously (Rajnovic et al., 2019). Briefly, 10 μL of filtrate was placed onto a nutrient agar plate containing 100 μL of host bacteria (OD₆₀₀ = 0.4) in 0.7 % agar. After clear zones (plaques) appeared, individual plaques were picked using sterile toothpicks and resuspended in 10 mL of fresh mid-log phase bacterial culture (OD₆₀₀ = 0.4). The resulting suspension was then subjected to a double-layer agar assay for plaque purification. After incubation overnight at 37 °C, the plaques were counted; then, a single clear plaque was isolated via double-layer agar plating, which was repeated three times.

We amplified phage vB_EcoS_P78 on a large scale by adding 1 mL of purified phage lysate to 100 mL of mid-log phase bacterial culture. The mixture was then incubated at 37 °C with shaking at 220 rpm until obvious bacterial lysis was observed. Bacterial cells were pelleted by centrifugation at 10,000 × *g* for 20 min. Phages in the supernatant were precipitated with PEG: solid PEG 8000 was added to a final 10 % (w/v) concentration, the mixture was shaken slowly, and then incubated overnight at 4 °C. The precipitated phages were collected by centrifugation (12,000 × *g*, 10 min, 4 °C) and were then resuspended in 2 mL of PBS (pH 7.2). The resuspension was filtered through a 0.22 μm sterile filter to remove impurities and obtain a clear phage suspension. The purified lysate was stored at 4 °C for later use.

### 2.3 Phage host range detection

The phage vB_EcoS_P78′s host range was examined employing the spot test method against 8 clinically isolated strains, which include 5 ESBL (extended-spectrum β-lactamases)-positive strains and 3 MDR (multidrug-resistant) strains, both of which are key multidrug-resistant *E. coli* (hybrid pathotypes). Additionally, a combination of phenotypic detection and genotypic verification was applied in the resistance confirmation method. Relevant research details were obtained by referring to Li et al. (2024). Briefly, 100 μL of each test strain (at a concentration of 1 × 10^8^ CFU/mL) was spread on LB Agar plates and dried at RT for 15 mins. Then, 10 μL from the serial diluted phage suspensions (1 × 10^10^ PFU/mL) was spotted on to bacterial colonies. After incubation at 37 °C overnight, bacterial susceptibility to being destroyed by phages could be judged by whether there were clear plaques.

### 2.4 Phage thermal and pH stability assay

To study the thermal stability of phage vB_EcoS_P78, we took 10 μL of phage suspension (10^10^ PFU/mL), and left it in tubes for two hours at temperatures of 4 °C, 28 °C, 37 °C, 50 °C, 60 °C, or 80 °C. After the completion of incubation of samples under all temperature conditions, 10 μL was diluted (10 times) serially with 90 μL water and then plated on the plates containing a pre-seeded LB medium with the host bacterium *E. coli* A78 for phage titer estimation. For the pH stability assay, phage aliquots were incubated in SM buffer (50 mM Tris-HCl, 100 mM NaCl, 8 mM MgSO_4_, 0.01% gelatin, pH 7.5) in a gradient of pH (3-12) for 2 hours. The titer of the survived phages after incubation was measured with the same spot assay method. All experiments were done with 3 independent replicates, and all assays were done in triplicate.

### 2.5 Determination of multiplicity infection (MOI)

With a few modest adjustments, the optimal multiplicity of infection (MOI) was calculated as previously reported (Zhang et al., 2023). To put it briefly, host bacterium *E. coli* A78 was cultured in LB broth to mid-exponential phase (OD₆₀₀ = 0.4) and adjusted to a final concentration of 10⁸ CFU/mL. To test target MOIs (100, 10, 1, 0.1, 0.01, 0.001), 100 μL of serially diluted phage lysate (10^5^–10^10^ PFU/mL) was mixed with 100 μL *E. coli* culture. Each mixture was supplemented with 800 μL fresh LB broth (total volume 1 mL) and incubated at 37 °C with 220 rpm shaking for 5 h. After incubation, phage titers in lysates were measured via double-layer agar assay. The MOI that produced the highest phage titer was defined as optimal, and all MOI conditions included three biological replicates.

### 2.6 One-step growth assay

The One-step growth experiments were conducted as previously described with minor modifications (Kropinski, 2018). Briefly, *E. coli* A78 cells in mid-exponential phase (OD_600_ = 0.4, 10^8^ CFU/mL) were exposed to bacteriophage suspensions at an MOI of 1.0 for 5 min at room temperature to facilitate adsorption. Unadsorbed phages were removed by triple centrifugation washes (10,000 g, 30 s). Washed complexes were resuspended in fresh LB, incubated at 37 °C with 220 rpm shaking, and 5-min aliquots were treated with 1 % chloroform to lyse cells for plaque enumeration via double-layer agar. Latent period was the time from adsorption completion at *t* = 0 to the first significant increase in extracellular phage titer (*p* < 0.05). Burst size was calculated as the ratio of maximal progeny phage yield to initial infected cell count. All experiments included triplicate biological replicates with data presented as mean ± SD.

### 2.7 Transmission electron microscopy (TEM) imaging

Phages were immobilized on 200-mesh steel grids with 2–10 nm carbon layers. Approximately 5 μL of the phage suspension (10^10^ PFU/mL) was placed on the grid and allowed to adsorb for 10 min. Excess liquid was blotted out with absorbent filter paper, and the sample was then dried again on fresh filter paper (1 min). Then 2 % uranyl acetate was used for negative staining on the samples for 90 s. Excess stain was removed, the grid was washed with ultrapure water, and the grid was dried under filter paper for 3 h. Phages were examined by transmission electron microscopy (TEM), provided by Hitachi High Technologies, Japan, at 80 kV. Morphological classification was performed following the guidelines set forth by ICTV (Turner et al., 2025).

### 2.8 Phage in vitro antibacterial assay

Centrifuge EHEC (control group) bacterial suspension during log stage, then wash the bacterial cells with PBS for 3 times. Then take the bacterial pellet and resuspend it in 100 μL of LB medium so that the final concentration is 1 × 10^6^ CFU/ mL. For the experimental group (Phage+ Bacteria), mix each bacterial suspension with 100 μL of a different phage or combination of phages in 96-well microplates at an MOI of 1.0. Place the microplates in a microplate reader and incubate them at 37 °C for 6 h. Read the OD₆₀₀ value of the mixture, and take OD₆₀₀ readings every 1 h for 6 h. For the control group, add only LB medium to the bacterial solution, and set up three parallel experiments.

### 2.9 Phage DNA extraction and whole genome sequencing

Genomic DNA from high titer phage lysate (1 × 10^10^ PFU/mL) was isolated. Phage enzymatic digestion and organic genomic DNA preparation were performed. In brief, 1 μL of phage suspension was incubated with DNase I (20 U) 10 μL and RNase A (20 mg/mL) 4 μL at 37 °C for 1 h to digest host DNA and RNA. Then 30 mL of 20 % SDS and 10 mg/mL proteinase K were added, and the mixture was incubated for 2 more hours at 37 °C to lyse phage and degrade proteins. Phage DNA was purified with phenol-chloroform extraction and DNA precipitation with anhydrous alcohol (Sambrook and Russell, 2006). Phage DNA concentration was quantified using a Qubit 2.0 Fluorometer with the dsDNA High Sensitivity Assay Kit (Thermo Fisher Scientific). DNA library was prepared from 1 ng of input DNA using the Nextera XT DNA Library Preparation Kit (Illumina, San Diego, CA, USA) according to the manufacturer’s protocol. Whole-genome sequencing (WGS) was conducted in paired-end mode (2 × 150 bp) using an Illumina NovaSeq 6000 sequencing system (Illumina).

### 2.10 Bioinformatics analysis

Raw bacteriophage sequencing reads were inspected for quality using FastQC (v0.12.1). Afterward, adapter sequences and low-quality bases were removed with Trimmomatic (v0.36) (Bolger et al., 2014). The de novo assembly of high-quality reads was assembled using plasmidSPAdes v3.13.1 (Bankevich et al., 2012) with k-mer size = 115. Putative open reading frames were predicted using Glimmer v3.02b on the assembled genome (Delcher et al., 2007). The prediction of transfer RNA (tRNA) genes was carried out using tRNAscan-SE (version 2.0) (Chan and Lowe, 2019). The complete phage genome sequence was searched against the Virulence Factor Database (VFDB) for virulence genes (Zhou et al., 2025), and antibiotic resistance genes were aligned with the antibiotic and resistance-related information housed in the Comprehensive Antibiotic Resistance Database (CARD) (Alcock et al., 2023). Finally, the predicted proteins were submitted for homology-based sequence searches against the NCBI RefSeq nr protein database and the Prokaryotic Virus Remote Homologous Groups (PHROG) database to functionally annotate the predicted genes (Terzian et al., 2021). The circular genome map of bacteriophage vB_EcoS_P78 was generated and visualized using CGview (Stothard et al., 2019).

The complete genome of vB_EcoS_P78 was blasted against the genome sequences of characterized bacteriophages in the NCBI GenBank database using BLASTn for homology search (Altschul et al., 1990), and next the calculation of pairwise intragenomic similarity for vB_EcoS_P78 and its closest relatives was performed using VIRDIC (Moraru et al., 2020). Phylogenetic Reconstruction: The amino acid sequences corresponding to the terminase large subunit and major capsid protein were aligned with MAFFT (v7.407) (Katoh and Standley, 2013) and the l-INS-i algorithm. Modelfinder was used to select among 546 candidate amino acid substitution models (Kalyaanamoorthy et al., 2017), with the optimal one determined by comparing their Bayesian information criterion (BIC) values. Here an ML tree was built based on the best-fitted model using IQ-TREE (v2.0) (Minh et al., 2020), using branch support calculated from 1000 bootstrap replications. The final phylogenetic tree was made and annotated using Interactive Tree of Life (iTOL) (Letunic and Bork, 2024). The structural conservation of genomic architectures was also analyzed via *Easyfig* (v2.2.5) synteny analysis (Sullivan et al., 2011).

### 2.11 Statistical analysis

Statistical analysis was conducted for this experiment via GraphPad Prism10.0 software. Moreover, One-way ANOVA was implemented, and differences reached statistical significance at the *P* < 0.05 level.

## 3 Results

### 3.1 Morphological features of vB_EcoS_P78

**Fig. 1:**
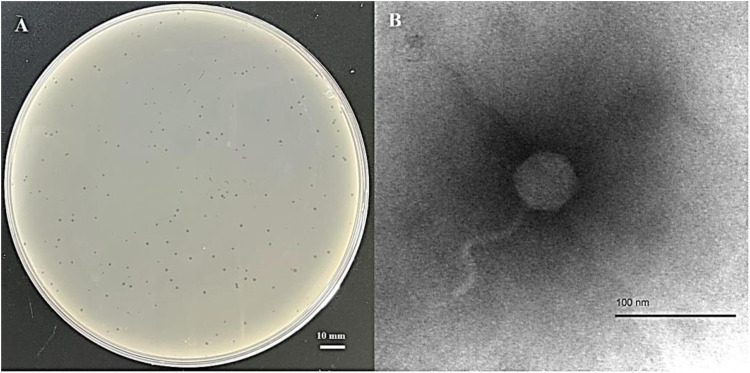
Morphology of vB_EcoS_P78. (A) Plaque morphology in a 9 cm diameter petri dish. (B) TEM images of vB_EcoS_P78. The scale bars represent 100 nm (bottom).

*Escherichia coli* phage vB_EcoS_P78 was isolated from swine feces using *E. coli* A78 as the host strain for enrichment. Plaque assay on double-layer agar (0.7% top agar) yielded clear plaques; additionally, the lytic phenotype of phage vB_EcoS_P78 was characterized via this assay. Overnight incubation at 37 °C resulted in the formation of circular plaques, with their average diameter being around 4 mm (Fig. 1A). Transmission electron microscopy (TEM) revealed that vB_EcoS_P78 possesses a head with a diameter of 47 ± 1 nm and a tail measuring 97 ± 4 nm in length (Fig. 1B).

### 3.2 Host range determination of vB_EcoS_P78

To determine the host range of phage vB_EcoS_P78, this study tested a panel of 8 *E. coli* strains. These strains were all isolated from the lung tissues of dead pigs infected with combined diarrhea and extraintestinal *E. coli*, and included two key types of multidrug-resistant *E. coli*: 5 ESBL-positive strains and 3 MDR (multidrug-resistant) strains. Spot tests showed that 6 of 8 (75 %) strains were susceptible to lysis and formed plaques on double-layer agar plates (Table 1). The susceptible strains spanned multiple serogroups, including O1, O3, O8, O11 and O55, covering common pathogenic serotypes in swine herds. Whole genome sequencing of these isolates revealed that they belonged to sequence types ST58, ST354, ST10 and ST542, which are associated with diarrhea-related sequence types.

All these pathogenic bacteria are multidrug-resistant *E. coli* strains and genomic sequences have been uploaded to the NCBI GenBank database with the project number PRJNA1044843.

### 3.3 Temperature stability test

**Fig. 2:**
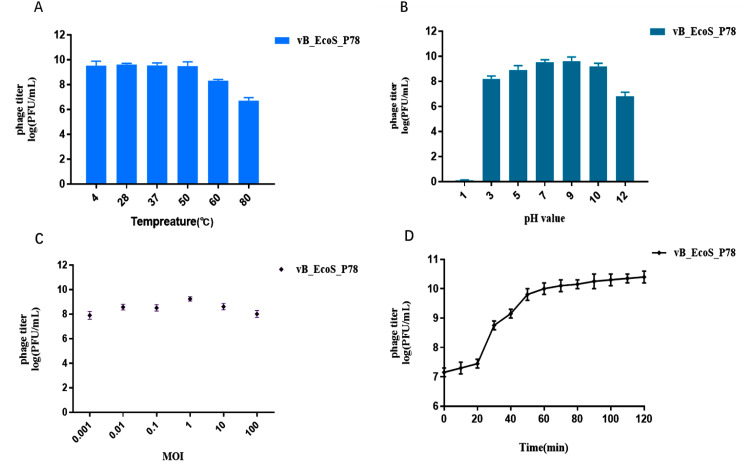
Biological characteristics of vB_EcoS_P78. Thermostability (A). The vertical axis shows the titre of the vB_EcoS_P78 in relation to the average titre at 4 °C, while the horizontal axis shows the temperature in degrees Celsius. (B) Stability of pH. The titre of vB_EcoS_P78 is displayed on the vertical axis, while the pH value is represented on the horizontal axis. (C) The optimal MOI curve. The coincubation times of *E. coli* A78 and vB_EcoS_P78 at various MOIs are shown on the horizontal axis. The standard deviation is shown by the error bars. (D) One-step growth curve.

The thermal stability of phage vB_EcoS_P78 was evaluated after 2 h of incubation at various temperatures (Fig. 2A). After incubation at temperatures ≤ 50 °C, the phage retained the highest titer with good stability, while phage titer began to drop moderately at 60 °C; viable phages, nonetheless, remained detectable at 80 °C, demonstrating strong physiological thermostability.

### 3.4 pH stability test

The pH stability test revealed that phage vB_EcoS_P78 retained high viability, with over 92.5 % of particles remaining infectious after 2 h of incubation within the pH range of 5.0 to 10.0 (Fig. 2B). A moderate reduction in titer was observed at pH 3.0. In contrast, a significant decrease occurred at pH 12.0, with only 70.8 % viability remaining. Complete inactivation was observed at pH 1.0, indicating irreversible capsid denaturation under extreme acidic conditions.

### 3.5 Optimal multiplicity of infection

The optimal multiplicity of infection was determined by infecting host bacteria with phage vB_EcoS_P78 at different MOI (0.001–100) followed by 5-h incubation. An MOI of 1.0 yielded the maximum phage titer (4 × 10^10^ PFU/mL), significantly exceeding other MOI conditions (Fig. 2C).

### 3.6 One-step growth curve

The one-step growth curve revealed a 20-min latent period followed by a 40-min rise phase (20–60 min post-infection), culminating in plateau establishment (Fig. 2D). Phage vB_EcoS_P78 exhibited a burst size of 220 PFU per infected cell. This demonstrates that vB_EcoS_P78 shows efficient replication, supporting strong bacterial clearance potential with proper dosing. Its short latent period enables rapid lysis and propagation, key for in vivo active infection control, while the high burst size sustains antimicrobial activity across cycles, reducing bacterial burden without high initial doses.

### 3.7 Phage in vitro lytic test

**Fig. 3:**
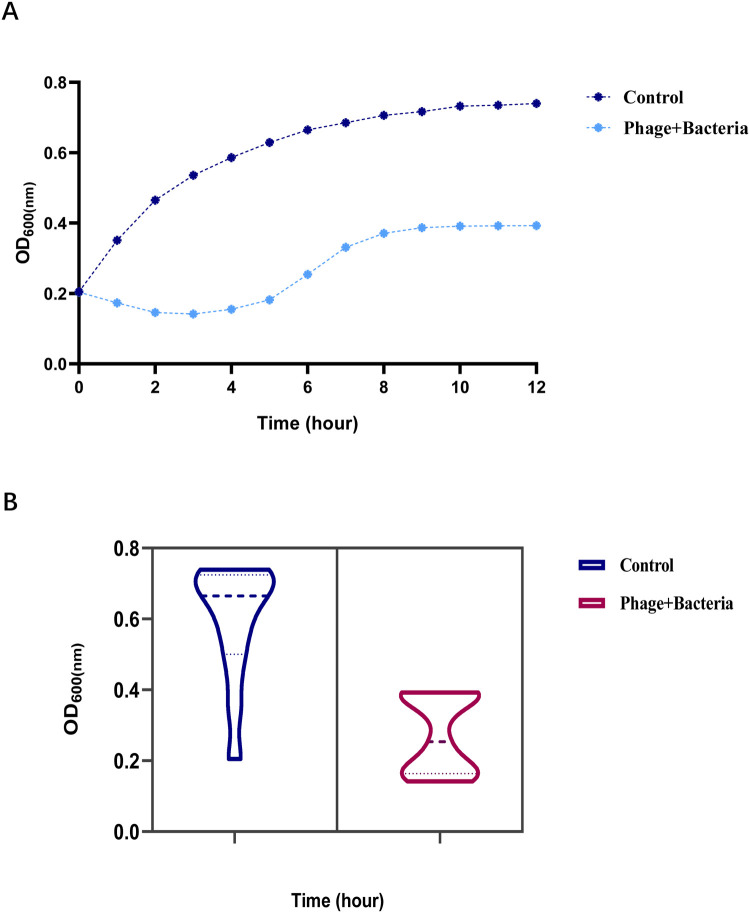
Bacterial reduction assay in vitro. Effect of vB_EcoS_P78 on the growth of EHEC compared with the control (A) Time - dependent growth curves of control group EHEC and EHEC co - cultured with phage vB_EcoS_P78. The abscissa (x-axis) is time (unit: h), used to observe dynamic changes in bacterial growth over time. The ordinate (y-axis) is absorbance at 600 nm (OD value), which indirectly reflects bacterial concentration. (B) A violin plot showing the bacterial density distribution of control group EHEC and EHEC co - cultured with phage vB_EcoS_P78 at OD₆₀₀. The plot for EHEC exhibits relatively high OD values with distribution concentrated in the high-value region; that for vB_EcoS_P78 shows relatively low OD values with distribution concentrated in the low-value range.

This study further evaluated the antibacterial potential of phage vB_EcoS_P78 against bacterial infections caused by diarrheagenic *E. coli*. Under in vitro culture conditions, it specifically investigated the phage's inhibitory effect on the growth of Enterohemorrhagic *E. coli* (EHEC). The bacteriostatic curve showed that the OD₆₀₀ value of the control group (EHEC) rose markedly and rapidly over time, reaching nearly 0.8 at around 12 h (Fig. 3A). In contrast, the OD₆₀₀ value of the Phage+ Bacteria group increased much less throughout the process, stabilizing at about 0.4 finally (Fig. 3B). This indicates that phage vB_EcoS_P78 significantly inhibits EHEC growth and restricts bacterial concentration increase. We performed three biological replicates, and the presented data are derived from the mean values of these replicates. The results demonstrate that phage vB_EcoS_P78 can effectively inhibit EHEC growth under in vitro culture conditions, providing important reference for subsequent studies on using phages to prevent and treat EHEC - induced bacterial infections.

### 3.8 Genome features of vB_EcoS_P78

**Fig. 4:**
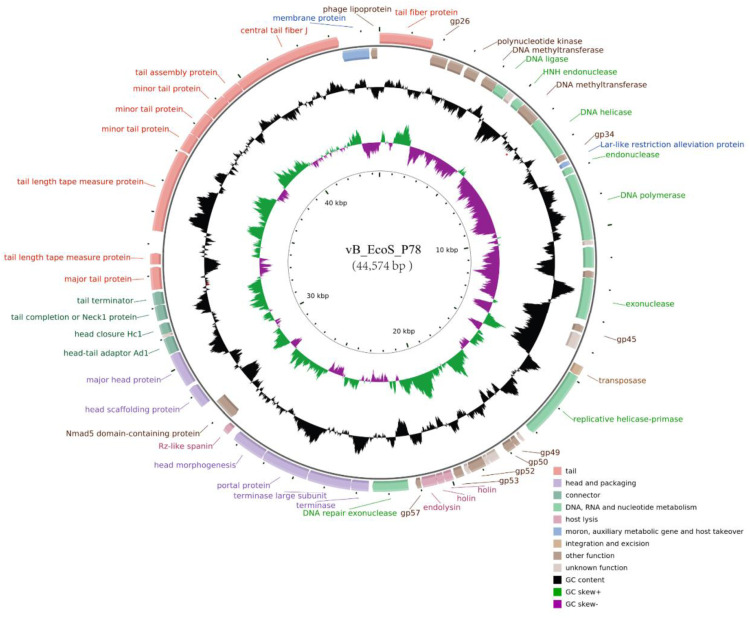
Genome overview of vB_EcoS_P78. Showing information about its genome structure and function. Different colors represent various types of functional modules in the genome overview. Additionally, key functional genes within each module are labeled along the map for clear identification, and the genomic features like GC content and GC skew are visualized through distinct inner ring tracks.

The complete genome of phage vB_EcoS_P78 is a circular double-stranded DNA molecule of 44,574 bp, with a GC content of 54.72 %. De novo assembly resulted in a single contig with an average coverage depth of approximately 16,966 X, supporting a high-confidence, gap-free genome sequence. In genome annotation, 58 ORFs were identified, comprising 36 open reading frames (ORFs) located on the positive strand and 22 ORFs situated on the negative strand. Thirty-five ORFs encode well-characterized proteins (35/58, 60.34 %), among which 23 ORFs (23/58, 39.66 %) are annotated as hypothetical proteins (8 ORFs) or putative conserved phage proteins of unknown functions (15 ORFs). No tRNAs were found via tRNAscan-SE, and no antibiotic resistance or virulence genes were detected in the phage genome. The entire genomic organization was viewed with CGView (Fig. 4).

Functional annotation supported a modular genome architecture, with distinct clusters encoding: structural components, including head and packaging (6 ORFs), connector (4 ORFs), and tail complexes (9 ORFs); nucleic acid metabolism, replication, and recombination (9 ORFs); and a lysis system (4 ORFs) consisting of an endolysin (vB_EcoS_P78_0033) that degrades the peptidoglycan layer of the host cell wall, two holins (vB_EcoS_P78_0031 and vB_EcoS_P78_0032) that form pores in the host cell membrane to facilitate endolysin translocation (Cahill and Young, 2019), and an Rz-like spanin (vB_EcoS_P78_0040) that disrupts the host outer membrane. These four components synergistically mediate the lysis of host cells, which is essential for phage progeny release (Chen et al., 2023).

### 3.9 Phylogenetic analysis of vB_EcoS_P78

**Fig. 5:**
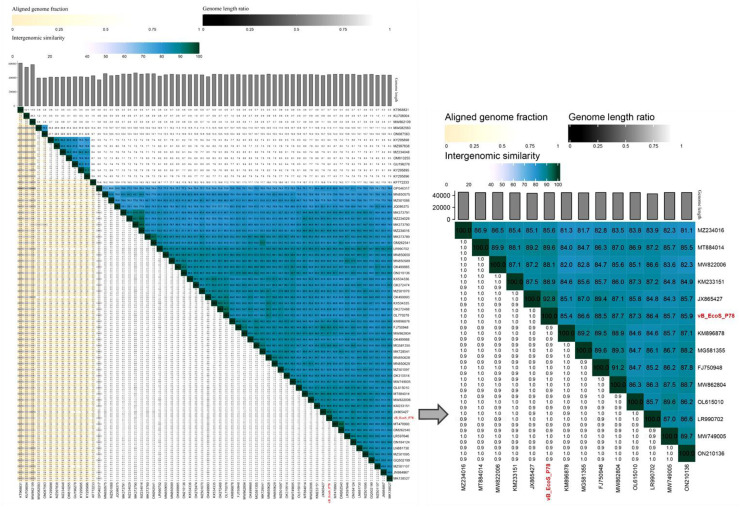
VIRIDIC heatmap of vB_EcoS_P78 and its closely related phages. The upper right section displays the inter-genomic similarities of phage pairs, with color intensity correlating to the degree of similarity. The part located in the lower left half sets out the relevant percentage data, which are the percentage coverage of phage one, the percentage alignment related to this phage, and the percentage coverage that applies to phage two.

Phylogenetic analysis of vB_EcoS_P78 was conducted to assess its taxonomic position. We first performed intergenomic similarity analysis using VIRIDIC showed that vB_EcoS_P78 shares the highest similarity (92.8 %) with *Dhillonvirus* phage *Escherichia* phage JL1 (Genbank Accession No.: JX865427), which is below the 95 % species demarcation threshold recommended by ICTV. Similarities with phages outside the genus *Dhillonvirus* were all below 70 % (shares the highest similarity of 17.4 % with Edwardsiella phage eiAU from Eiauvirus genus and the lowest similarity of 0.7 % with *Pseudomona*s phage YMC11/02/R656 from *Bugaksanvirus* genus), supporting its classification within this genus (Fig. 5).

**Fig. 6:**
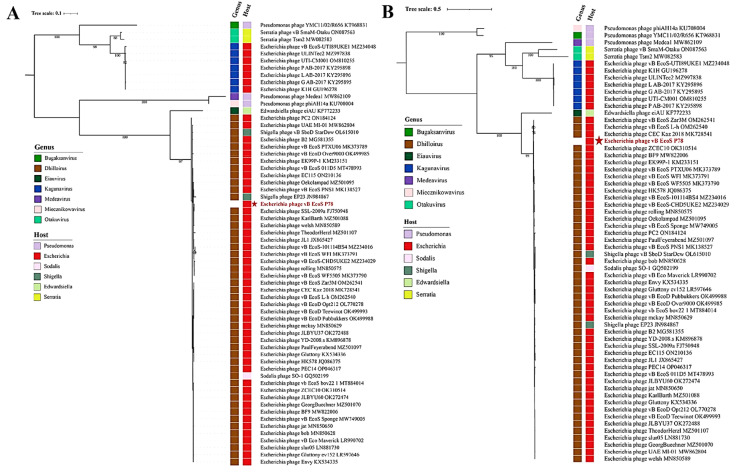
Phylogenetic trees of phage vB_EcoS_P78 based on key protein sequences. Phylogenetic trees based on (A) the major capsid protein (MCP) and (B) the terminase large subunit (TerL) protein sequences. The phage vB_EcoS_P78 is highlighted in red to emphasize its evolutionary placement. Bootstrap values ≥ 70 % are indicated at corresponding nodes, and the trees support the taxonomic classification of phage vB_EcoS_P78 within the genus *Dhillonvirus*.

**Fig. 7:**
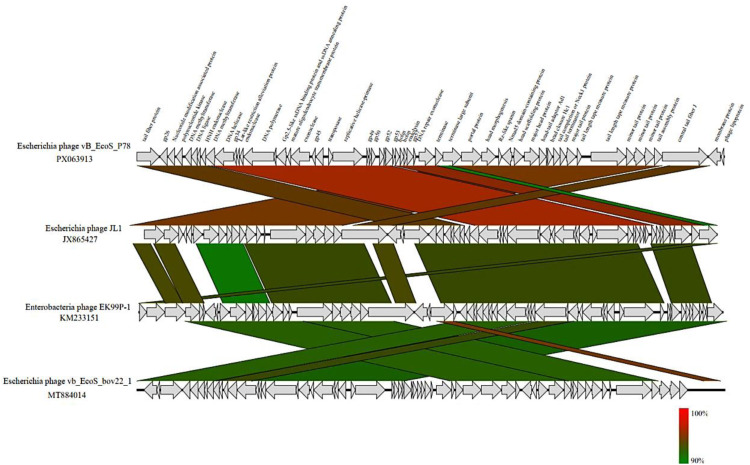
Visualization of Clinker-Mediated Gene Cluster Comparison Between Phage vB_EcoS_P78 and Its Phylogenetically Closest Relative. The Clinker gene cluster comparison of the whole genome of phage vB_EcoS_P78 against its closest relative phage was visualized using a similarity-based color scheme: red connecting lines indicate high percent nucleotide identity, whereas green connecting lines denote lower sequence identity.

To further resolve the evolutionary position of vB_EcoS_P78, maximum-lielihood phylogenetic trees were constructed based on the amino acid sequences of two essential viral proteins: the major capsid protein (MCP) and the terminase large subunit (TerL). The MCP-based phylogeny (Fig. 6A) demonstrated that vB_EcoS_P78 forms a distinct clade with other members of the genus *Dhillonvirus*, supported by a high bootstrap value of 97 %. Similarly, the TerL-based phylogeny (Fig. 6B) placed vB_EcoS_P78 within the *Dhillonvirus* clade with even stronger statistical support (bootstrap value of 99 % for TerL, 97 % for MCP). The consistent topological placement in two independent phylogenetic analyses, based on proteins with different functions and evolutionary rates, provides robust evidence for classifying vB_EcoS_P78 as a member of the genus *Dhillonvirus*. Moreover, comparative genomic analysis revealed significant synteny between vB_EcoS_P78 and *Escherichia* phage JL1, with conserved gene order and orientation across core functional modules (Fig. 7).

## 4 Discussion

### 4.1 Phage vB_EcoS_P78 infection characteristics and biological significance in multidrug -resistant *E. coli*

Bacteriophages, as viruses that parasitize bacteria, hold potential application prospects as an alternative to antibiotics. In this study, a lytic bacteriophage, designated *E. coli* phage vB_EcoS_P78, was isolated from swine feces using *E. coli* A78 as the host strain for enrichment. The phage produced distinct, clear, circular plaques approximately 4 mm in diameter after overnight incubation at 37 °C on double-layer agar plates. Viral particles visualized through transmission electron microscopy exhibited an icosahedral head and an extended non-contractile tail, with morphological attributes corresponding to members of the *Siphoviridae* family in the *Caudoviricetes* class. Intergenomic similarity analysis showed that vB_EcoS_P78 has the highest similarity with *Escherichia* phage JL1, a member of the genus *Dhillonvirus*, suggesting that vB_EcoS_P78 may represent a novel species within this genus (Turner et al., 2025).

Notably, vB_EcoS_P78 demonstrates the ability to infect multiple ESBL-producing and MDR *E. coli* strains spanning a range of serogroups and sequence types. This broad host range appears to be a conserved trait within the *Dhillonvirus* genus. For instance, *Escherichia* phage vB_EcoS_PS2 was reported to exhibit high lytic specificity against diverse *E. coli* serotypes, including O157:H7, O6, and O78:K80:H12 (Zhang et al., 2025), which may result from the recognition of highly conserved bacterial surface receptors - such as core lipopolysaccharide (LPS) structures or outer membrane proteins -across diverse *E. coli* lineages. This ability to target multiple serotypes and sequence types is of particular therapeutic importance, as it enhances the potential utility of *Dhillonvirus* phages in cocktail formulations aimed at combating ESBL-producing *E. coli* infections (Pfeiffer and Virgin, 2016). Further studies focusing on receptor binding specificity and genomic comparisons of host recognition modules within this genus could elucidate the molecular mechanisms underpinning their broad infectivity and support the rational design of phage-based therapeutics.

The biological characteristics of vB_EcoS_P78 further underscore its promise as a therapeutic agent. It maintains activity over a wide temperature and pH range, indicating a certain level of stability and environmental adaptability, which supports its suitability for pharmaceutical development and storage (Malik et al., 2017). The phage also exhibits relatively efficient replication capacity, including a short latent period and strong lytic ability. The brief latent period enables rapid initiation of lysis and swift propagation within bacterial populations, a critical feature for controlling active infections in vivo (Zhang et al., 2023). Besides, its high burst size ensures the sustained exertion of antibacterial activity throughout multiple infection cycles, thereby achieving effective bacterial clearance (Zhang et al., 2023). In addition, the phage vB_EcoS_P78 in this study has shown promising application potential in preventing diarrhea caused by enterohemorrhagic *Escherichia coli* (EHEC). In vitro bacteriostatic results indicated that phages can effectively inhibit the growth of EHEC pathogens and also exhibit good lytic activity against other clinically isolated multidrug-resistant *Escherichia coli* strains associated with diarrhea. Therefore, the phage provides a novel and highly targeted candidate strategy as well as experimental evidence for the clinical prevention of diarrhea caused by EHEC and MDR *E. coli* (Strathdee et al., 2023).

### 4.2 Insights from genomic analysis: functional traits and features of lytic modules of phage vB_EcoS_P78

Genomic analysis provides crucial insights into the safety and functional capacity of vB_EcoS_P78. The absence of genes encoding antibiotic resistance, virulence factors, or lysogeny-related proteins addresses important safety concerns for therapeutic application. The modular genome organization, with distinct clusters for structural proteins, DNA metabolism, and host lysis, is consistent with typical bacteriophage architecture but shows specific adaptations that merit further investigation (Sada et al., 2024). The presence of a complete lysis module (endolysin, two holins, and spanin) is particularly relevant, as these components work synergistically to ensure efficient host cell lysis and progeny release (Roach et al., 2017).

Regarding structural genes unique to vB_EcoS_P78, the identified endolysin, dual holins, and Rz-like transmembrane protein form a complete lytic system, which is distinct from most single-holin-containing phages targeting MDR *E. coli*. This unique combination may enhance its efficiency in disrupting the host cell envelope, a potential structural basis for its lytic activity against MDR strains (Cahill and Young, 2019). For receptor-binding insights, although direct receptor-binding protein (RBP) identification requires further experiment, the unique lytic gene cluster implies potential coordination between its lytic machinery and receptor recognition, and this speculation is supported by previous studies showing that holin-mediated membrane permeabilization can assist RBPs in accessing host receptors (Wang et al., 2000). This combination of dual holins alongside the endolysin and Rz-like protein is not commonly reported in characterized *Dhillonviruses*, where single holin-encoding genes are more typical (Vasileiadis et al., 2024). The presence of two functionally distinct holins may enhance membrane permeabilization efficiency, potentially contributing to its robust lytic activity against multidrug-resistant *E. coli*, a feature that sets it apart from known members of the genus.

## 5 Conclusion

In summary, this study comprehensively characterized a novel bacteriophage, *Escherichia* phage vB_EcoS_P78. The phage has typical siphovirus morphology, lytic activity against ESBL-producing and multidrug-resistant *E. coli*, and strong environmental stability. Intergenomic similarity and phylogenetic analysis assigned it to the genus *Dhillonvirus*. Its genome lacks virulence and antibiotic resistance genes, supporting its safety for potential therapeutic applications. However, limitations are still present: host range detection has not been extended to a larger panel of clinical *E. coli* isolates, leaving its lytic spectrum for diverse clinical strains unclear; additionally, in vivo data including efficacy and safety evaluations in animal infection models are lacking, and addressing these two aspects in follow-up studies will be critical to advancing its translational value. Overall, vB_EcoS_P78 is a promising candidate for developing phage-based therapies against multidrug-resistant bacterial infections.

## Nucleotide sequence number

The complete genome sequence corresponding to phage vB_EcoS_P78 was submitted to the GeneBank database, and it has been assigned the accession number to facilitate its location and access.

## Author statement

All authors have made substantial contributions to the work and have approved the final version of the manuscript. We confirm that the manuscript has not been published previously and is not under consideration for publication elsewhere. There are no any conflicts involved in the manuscript.

Rong Wen: Devised the study concept and design, and created the preliminary draft of the manuscript. Jiahao Tong: executed the experiments, including sample collection, data acquisition, and lab analyses. Shoude Liu and Chengbo Zheng: handled statistical analysis and result interpretation. Donghai Peng and Jinshui Zheng: Jointly guide team members in conducting experimental operations, data collection, and preliminary analysis to ensure the standardization of research methods and the reliability of data. Ming Sun: Conduct critical revision of the work to evaluate its intellectual merit assessing ideas’ originality, reasoning’s rigor, and contributions’ significance to the field. All authors reviewed and approved the final version, and collectively accept accountability for investigating and resolving any concerns related to the work’s accuracy or integrity.

## CRediT authorship contribution statement

**Rong Wen:** Writing – original draft, Conceptualization. **Jiahao Tong:** Methodology, Investigation. **Shoude Liu:** Visualization, Data curation. **Chengbo Zheng:** Visualization, Data curation. **Jinshui Zheng:** Validation, Supervision. **Donghai Peng:** Validation, Supervision. **Ming Sun:** Writing – review & editing, Resources, Project administration.

## Declaration of competing interest

The final version was examined and approved by all authors, who also take joint responsibility for looking into and addressing any issues with the accuracy or integrity of the work.

## Data Availability

Data will be made available on request.

## Appendix Supplementary materials

Image, application 1
